# Diurnal Variations in Vascular Endothelial Vasodilation Are Influenced by Chronotype in Healthy Humans

**DOI:** 10.3389/fphys.2019.00901

**Published:** 2019-07-10

**Authors:** Elise R. Facer-Childs, Katie Pake, Vivian Y. Lee, Samuel J. E. Lucas, George M. Balanos

**Affiliations:** ^1^School of Sport, Exercise and Rehabilitation Sciences, University of Birmingham, Birmingham, United Kingdom; ^2^School of Psychology, University of Birmingham, Birmingham, United Kingdom; ^3^Centre for Human Brain Health, University of Birmingham, Birmingham, United Kingdom; ^4^Turner Institute for Brain and Mental Health, School of Psychological Sciences, Monash University, Melbourne, VIC, Australia

**Keywords:** chronotype, sleep, flow-mediated dilatation, vascular endothelial function, diurnal variation, cardiovascular regulation

## Abstract

**Introduction:** The time of day when cardiovascular events are most likely to occur is thought to be aligned with the circadian rhythm of physiological variables. Chronotype has been shown to influence the time of day when cardiovascular events happen, with early chronotypes reported to be more susceptible in the morning and late chronotypes in the evening. However, no studies have investigated the influence of chronotype on physiological variables responsible for cardiovascular regulation in healthy individuals.

**Methods:** 312 individuals completed the Munich ChronoType Questionnaire to assess chronotype. Twenty participants were randomly selected to continue into the main study. In a repeated-measures experiment, participants were tested between 08:00 and 10:00 h and again between 18:00 and 20:00 h. Measurements of mean arterial pressure, heart rate and vascular endothelial vasodilation via flow-mediated dilatation (FMD) were obtained at each session.

**Results:** Individual diurnal differences in mean arterial pressure and heart rate show no significant relationship with chronotype. Diurnal differences in FMD showed a significant correlation (*p* = 0.010), driven by a clear significant relationship in the evening and not the morning (*p* < 0.001).

**Conclusion:** These preliminary data indicate that chronotype influences the diurnal variation of endothelial vasodilation measured using flow-mediated dilatation. Furthermore, we show that the influence of chronotype is much stronger in the evening, highlighting an increased susceptibility for later types. These findings are consistent with the diurnal rhythm in cardiovascular events and uncover potential mechanisms of local mediators that may underpin the influence of chronotype in the onset of these events.

## Introduction

Humans exhibit a time-of-day pattern in the prevalence of cardiovascular events. There is a peak incidence of cardiovascular events such as AMI (Manfredini et al., 2013), sudden cardiac death (Muller et al., 1987; Cohen et al., 1997) and stroke (Elliott, 1998) in the morning. These time-of-day differences in incidence of cardiovascular events have been proposed to be influenced by circadian rhythms of critical physiological variables responsible for cardiovascular regulation. For example, BP has been reported to peak in the morning (Kario et al., 2003; White, 2007), whereas vascular reactivity has been reported to be attenuated in the morning. Thus, the alignment of these circadian rhythms creates a temporal window of excessive susceptibility, which increases an individual’s risk of a cardiovascular event in response to relatively minor stressors (Manfredini et al., 2013). However, a number of studies provide evidence to indicate that there is an additional secondary afternoon peak in the incidence of cardiovascular events such as cardiac death (Arntz et al., 1993), stroke (Stergiou et al., 2002), and cardiac arrests (Peckova et al., 1998). It is possible that individual differences (i.e., chronotype) in the circadian regulation of physiological function and reactivity may be partly influencing this separation in the prevalence of cardiovascular events.

Chronotype influences a large number of behavioral and physiological variables such as hormonal rhythms (Burgess and Fogg, 2008), athletic performance (Facer-Childs and Brandstaetter, 2015), and brain function (Facer-Childs et al., 2019). Therefore, it seems reasonable to hypothesize that individual differences also exist in the circadian rhythm of cardiovascular regulation. The distribution of chronotype within an experimental sample is likely to vary significantly, thus potentially explaining why there are discrepancies between studies. A pilot study by Selvi et al. (2011) illustrated that incidence of cardiovascular events is influenced by chronotype. These findings showed that peak onset of AMI for early chronotypes occurred in the morning whereas the peak in late chronotypes was later in the day. Further, in support of these epidemiological findings, evidence indicates that the circadian pattern of core body temperature is altered depending on chronotype (Bailey and Heitkemper, 2001) and has been linked to endothelial function (Amiya et al., 2013). This may result in different temporal windows of enhanced susceptibility to cardiovascular events thus provide an explanation as to why chronotype influences the time-of-day pattern in occurrences of such events.

The vascular endothelium plays a key role in cardiovascular homeostasis through the modulation of vascular tone in response to metabolic changes, inhibition of platelet adhesion and immune function. One of the functions of the endothelium is to release vasoactive factors [e.g., nitric oxide (NO), prostaglandins, and endothelial-derived hyperpolarizing factors] to regulate relaxation of vascular smooth muscle. These substances are also involved in the modulation of other functions such as inflammation and cell proliferation (Durand and Gutterman, 2013). Prolonged exposure to cardiovascular risk e.g., aging, can result in damage to endothelial cells and attenuated responses in the regulation of vascular tone leading to endothelial dysfunction (Deanfield et al., 2007). As mentioned previously, it is well known that circadian rhythms in BP and HR are controlled by biological clocks, with lesions to the suprachiasmatic nucleus resulting in arrhythmia (Lemmer, 2006). However, a number of animal studies have shown that this rhythmicity is not dependant on endothelial-derived NO (Arraj and Lemmer, 2006, 2007), suggesting that temporal variations in local mediators of endothelium-dependant vasomotor functions may be impacted via alternative mechanisms.

Flow-mediated dilatation (FMD) is a widely used, non-invasive technique to assess an index of macrovascular endothelial function. FMD acts as a surrogate marker for NO bioavailability through an increased blood flow following a period of ischemia causing endothelial cells to release vasoactive factors (e.g., NO), resulting in the relaxation of the vessel. The percentage change in the diameter of the vessel from baseline is used as an indicator of endothelial function, with bigger changes indicating better endothelial response and smaller changes indicating attenuated responses (Sandoo et al., 2010).

Against this background, this study aimed to identify the influence of chronotype on the diurnal variation of endothelial vascular vasodilation measured non-invasively using brachial ultrasonography. It was, therefore, hypothesized that there would be a significant effect of chronotype on vascular endothelial vasodilation and that time of day would be a significant predictor of attenuated responses in FMD measurements.

## Materials and Methods

### Participants

All procedures involving human participants were carried out in accordance with the all relevant regulatory standards. The protocol was approved by the University of Birmingham Research Ethics Committee. All participants gave written informed consent before involvement in accordance with the 1964 Declaration of Helsinki and its later amendments. All details provided were given on a voluntary basis and participants were free to withdraw at any time.

Assessment for chronotype using the Munich ChronoType Questionnaire (MCTQ) (Roenneberg et al., 2003) was performed in 312 healthy individuals. A subset of these participants were randomly selected based on mid-sleep times on free days (MSF_sc_) to represent the total population and were invited to take part in the main study. Power calculations in G^*^Power (Faul et al., 2007) were conducted using an alpha of 0.05 and a power of 0.90. An estimated sample size of *n* = 16 was calculated for an effect size of 0.77. Twenty participants agreed to take part, however three individuals dropped out prior to data collection. The final sample consisted of 17 participants (age 24.5 ± 5.3, BMI 23.9 ± 1.3, eight females). Participants were healthy, active individuals with no history of cardiovascular or respiratory disease. Females who were not on contraceptive medication were tested in the follicular phase of their menstrual cycle.

### Experimental Procedures

Participants were required to visit the laboratory on three occasions. Participants first visited the laboratory for a preliminary session during which they were familiarized with the experimental protocol and provided demographic information. The second visit (morning, AM) was conducted between 08:00 and 10:00 h and the third (evening, PM) between 18:00 and 20:00 h, both with identical experimental protocols. Room temperature was maintained at 21°C and light exposure was kept constant at each experimental session. On arrival, participants resting HR and BP were measured using Omron 705IT electronic BP monitor (Omron healthcare, Japan). Pre-test standardization included abstinence from exercise and caffeine for at least 12 h and food for 4 h prior to each experimental session. Participants were encouraged to follow normal sleep behavior, to wake up approximately 1 h prior to the morning test and limit exposure to natural light during day of testing. All participants traveled to the university by car and used the lift to access the laboratory to further control for exertion and exposure to natural light.

### Vascular Endothelial Vasodilation

Endothelial vascular vasodilation was measured using the technique of flow-mediated dilatation (FMD). An ultrasound transducer (Terason Usmart 3300, Teratech corporation, United States) was used to image the brachial artery and its diameter was measured automatically with the use of proprietary border detection software (Cardiovascular Suite v3.4.0, Quipu, Italy). A sphygmomanometric cuff was inflated to 200 mmHg for a period of 5 min to produce an adequate hyperaemia to allow flow-mediated dilatation without compromising participant comfort (Raitakari and Celermajer, 2000). Brachial artery diameter and blood flow measurements were made at baseline during cuff inflation and after a further 2 min following cuff deflation. Vascular endothelial function was measured as the diameter change of the brachial artery from baseline to peak dilatation following release of the cuff.

### Data Analysis

All data were statistically analyzed in GraphPad Prism (version 7.00) using paired *t*-tests and linear regression. Non-parametric tests were implemented where data did not follow a normal distribution. Results are reported as mean ± standard error of the mean apart from age and BMI which is reported as mean ± standard deviation. Absolute measurement values are given to one decimal place and statistical values are given to two decimal places apart from when *p* < 0.001 (ns, not significant).

## Results

**FIGURE 1:**
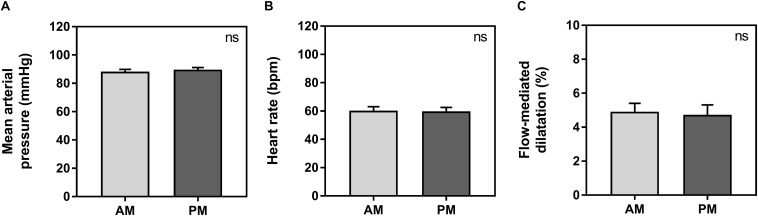
Average measurements between morning (AM, light gray) and evening (PM, dark gray) for mean arterial pressure **(A)**, heart rate **(B)**, and flow-mediated dilatation **(C)**. Significance is shown in the top right corner (ns, not significant).

Baseline vessel diameter between morning (3.8 ± 0.1 mm) and evening (3.8 ± 0.2 mm) was not significantly different (*p* = 0.66). There were no significant differences between morning and evening sessions for average HR, MAP or FMD measurements. Average HR was 60 ± 3 bpm in the morning and 60 ± 3 in the evening (*p* = 0.64). Average MAP was 88 ± 2 mmHg in the morning and 90 ± 1 mmHg in the evening (*p* = 0.19). Average FMD was 4.9 ± 0.5% in the morning and 4.7 ± 0.6% in the evening (*p* = 0.79) (Figure 1).

**FIGURE 2:**
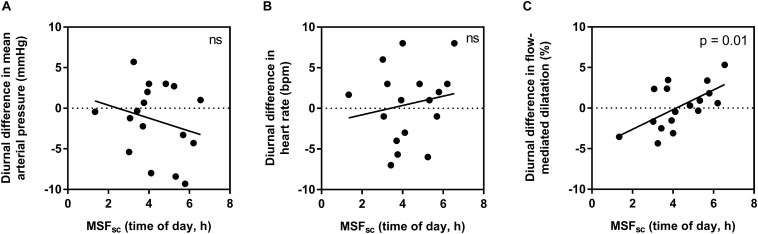
The influence of chronotype (using corrected mid-sleep times on free day, MSF_sc_) on individual diurnal differences in mean arterial pressure **(A)**, heart rate **(B)**, and flow-mediated dilatation **(C)**. Significance is shown in the top right corner (ns, not significant).

When assessing if individual diurnal variations (difference between morning and evening measurements) are influenced by chronotype, no significant relationships were found for HR (*R*^2^ = 0.03, *p* = 0.51) or MAP (*R*^2^ = 0.06, *p* = 0.34). However, a moderate-to-strong significant correlation was observed for FMD (*R*^2^ = 0.37, *p* = 0.01) (Figure 2).

**FIGURE 3:**
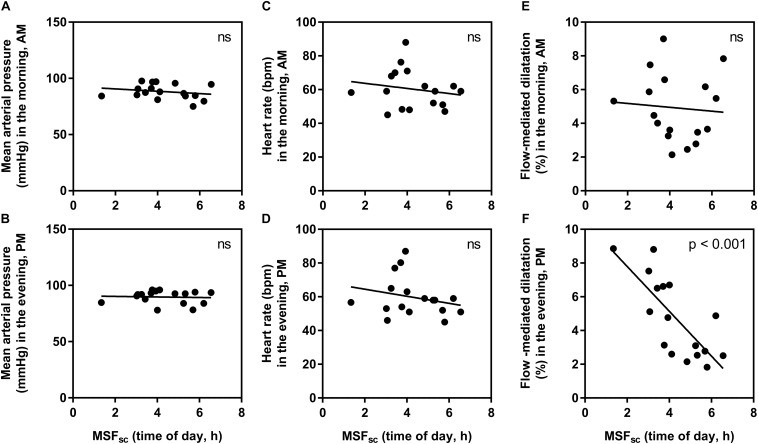
The relationship between chronotype (corrected mid-sleep time on free days, MSF_sc_) and absolute measurement values in the morning (AM) and evening (PM). **(A)** mean arterial pressure in the morning, **(B)** mean arterial pressure in the evening, **(C)** heart rate in the morning, **(D)** heart rate in the evening, **(E)** flow-mediated dilatation in the morning, **(F)** flow-mediated dilatation in the evening. Significance is shown in the top right corner (ns, not significant).

To explore this relationship further, the influence of chronotype on absolute values for each measurement in the morning and evening were explored separately (Figure 3). There were no significant relationships found at either time point for HR (morning: *R*^2^ = 0.03, *p* = 0.49, and evening: *R*^2^ = 0.06, *p* = 0.35) or MAP (morning: *R*^2^ = 0.05, *p* = 0.41, and evening: *R*^2^ < 0.01, *p* = 0.83). For FMD measurements, there was no significant influence of chronotype in the morning (*R*^2^ < 0.01, *p* = 0.77), but a strong and significant relationship observed in the evening (*R*^2^ = 0.58, *p* < 0.001).

## Discussion

Endothelium dependent vasodilation is thought to be a critical physiological variable involved in the incidence of cardiovascular events (Kubo et al., 1991). Compromised vascular endothelial function has previously been linked to an increased risk for the development of cardiovascular events and thus, different chronotypes may be exposed to increased risk for such events in the morning or the afternoon due to a corresponding diurnal pattern of vascular reactivity (Yeboah et al., 2009; Inaba et al., 2010).

In this study, we uncover an influence of chronotype on individual diurnal differences in vascular endothelial vasodilation measured using FMD, in a young healthy population. Those with earlier chronotype (earlier MSF_sc_) seemed to have an attenuated FMD response in the morning compared to the evening, while those with a later MSF_sc_ had an attenuated response in the evening compared to the morning (Figure 2). Interestingly, these diurnal differences were driven by an influence of chronotype on absolute FMD values during the evening (Figure 3). The observed morning attenuation in FMD supports previous findings (Etsuda et al., 1999; Elherik et al., 2002; Otto et al., 2004; Jones et al., 2010). However, attenuation in FMD in the evening has not yet been reported. The reason for this may be that previous literature does not consider the influence of chronotype on the diurnal rhythm of endothelial vasodilation. Since we observed no significant differences in average FMD between morning and evening when looking at the whole sample, this further highlights the need to account for chronotype when investigating diurnal variations in cardiovascular regulation (Figure 1). These findings support our hypothesis and provide a potential explanation for why earlier chronotypes may be more likely to suffer from an AMI in the morning, while later chronotypes are more likely to suffer from an AMI in the evening, as reported by Selvi et al. (2011). These findings also provide evidence to support the secondary diurnal peak in cardiovascular events, which appear to be driven by attenuated responses in individuals with a later chronotype. This proposes a possible role for chronotype in the development of cardiovascular risk, which could inform clinical considerations for the way patients are treated in order to mitigate these risks.

It is well known in the literature that BP surges in the morning 1–2 h following awakening and progressively declines throughout the day (Kario et al., 2003; White, 2007). This early morning surge in BP is thought to be associated with an increased α-sympathetic vasoconstrictor activity (Panza et al., 1991) and is considered to be one of the main cardiovascular risk factors responsible for cardiovascular events (Kario et al., 2003; White, 2007). In the current study there were no significant differences in the diurnal variation of MAP, which is in contrast to multiple other studies. However, it is possible that we missed the morning peak because the timing of the morning sessions (08:00 to 10:00 h) was too late. In addition, here we are studying young, healthy humans who would be expected to have good cardiovascular health. Repeated exposure to stressors throughout life causes reduced integrity of endothelial cells and increased susceptibility to cardiovascular risk. Based on our observations here, we suggest that young, healthy individuals are more sensitive to individual diurnal variations in local mediators of vascular regulation (FMD) compared to systemic measures such as BP and HR. This, in part, could be due to the relatively good cardiovascular health that would be expected of this cohort. As mentioned previously, animal studies have demonstrated that endothelial NO is not involved in the circadian regulation of BP or HR (Arraj and Lemmer, 2006, 2007), which is in line with the absence of diurnal variations in BP and HR found in this study. FMD is a measure of a local compensatory response to transient ischemia which was influenced by chronotype, and thus, individual diurnal differences in local mediators may be impacted by alternative mechanisms.

The main limitation of the current study is that although participants were clearly informed about standardization requirements for the period before the laboratory sessions adherence to these requirements was self-reported and therefore subjective. Another consideration for this study is that our results are based on a single day of measurements. This may have resulted in those who naturally wake up later on a normal day to have been compromised during both laboratory sessions. During the morning session they would have been awake earlier than normal, and assuming that they followed their habitual sleep time the previous night, during the afternoon session they would have been experiencing the effects of acute sleep restriction. However, since late chronotypes are often required to wake up earlier than their preferred time due to social-professional commitments, this design increases the external validity of the study.

## Conclusion

In summary, we show that chronotype influences the diurnal variation of endothelial vascular vasodilation. Earlier chronotypes demonstrated attenuated responses in the morning whereas for later chronotypes it was attenuated in the evening. Furthermore, the influence of chronotype on FMD was highly significant during the evening, driven by attenuated responses in later chronotypes. It could, therefore, be inferred that chronotypes exhibit altered windows of increased susceptibility to cardiovascular events due to differences in the alignment of physiological variables responsible for cardiovascular regulation. These findings provide a possible explanation for individual diurnal variations in local mediators of endothelial function, which may not be affected by the same circadian regulatory mechanisms observed in systemic measures such as BP and HR.

This study focused on the relationship between chronotype and an index of endothelial function, however chronotype has also been shown to influence the time-of-day differences in other variables such as cognitive (Blatter and Cajochen, 2007; Preckel et al., 2011) and physical performance (Tamm et al., 2009; Facer-Childs and Brandstaetter, 2015; Facer-Childs et al., 2018). Therefore, this study provides further incentive to broaden research in this area in order to establish the underlying physiological mechanisms that underpin the influence of chronotype on physiology and subsequent behavior. Overall, we provide a unique insight into the physiological differences between chronotypes, which have previously not been investigated, and may aid in identifying and managing when an individual is most at risk of a cardiovascular event.

## Ethics Statement

All procedures involving human participants were in accordance with the ethical standards of the University of Birmingham Research Ethics Committee and with the 1964 Helsinki declaration and its later amendments or comparable ethical standards. Participants gave written informed consent before involvement, all details provided were given on a voluntary basis and participants were free to withdraw at any time.

## Author Contributions

GMB and SJEL conceived and designed the study with the help of ERF-C. KP and VYL collected the data. ERF-C analyzed the data with inputs from GMB. All authors contributed to writing the manuscript.

## Conflict of Interest Statement

The authors declare that the research was conducted in the absence of any commercial or financial relationships that could be construed as a potential conflict of interest.

## Abbreviations

AMI: acute myocardial infarction
BP: blood pressure
FMD: flow mediated dilatation
HR: heart rate
MAP: mean arterial pressure
MSF_sc_: corrected mid-sleep on free days.
